# Changes in epigenetic profiles throughout early childhood and their relationship to the response to pneumococcal vaccination

**DOI:** 10.1186/s13148-021-01012-w

**Published:** 2021-02-04

**Authors:** Sara Pischedda, Daniel O’Connor, Benjamin P. Fairfax, Antonio Salas, Federico Martinon-Torres, Andrew J. Pollard, Johannes Trück

**Affiliations:** 1 Genetics, Vaccines and Infections and Pediatrics Research Group (GENVIP), Instituto de Investigación Sanitaria de Santiago, Santiago de Compostela, Spain; 2 Translational Pediatrics and Infectious Diseases, Department of Pediatrics, Hospital Clínico Universitario de Santiago de Compostela, Santiago de Compostela, Spain; 3 Hospital Clínico Universitario de Santiago (SERGAS), Unidade de Xenética, Instituto de Ciencias Forenses, Facultade de Medicina, Universidade de Santiago de Compostela, and GenPoB Research Group, Instituto de Investigaciones Sanitarias (IDIS), Galicia, Spain; 4 Oxford Vaccine Group, Department of Paediatrics, University of Oxford, and The NIHR Oxford Biomedical Research Centre, Oxford, UK; 5 MRC-Weatherall Institute of Molecular Medicine, University of Oxford, Oxford, UK; 6 Division of Immunology and Children’s Research Center, University Children’s Hospital Zurich, University of Zurich (UZH), Zurich, Switzerland

**Keywords:** Pneumococcal vaccination, Immune system, Childhood, Vaccine response, Epigenetics, DNA methylation

## Abstract

**Background:**

Pneumococcal infections are a major cause of morbidity and mortality in young children and immaturity of the immune system partly underlies poor vaccine responses seen in the young. Emerging evidence suggests a key role for epigenetics in the maturation and regulation of the immune system in health and disease. The study aimed to investigate epigenetic changes in early life and to understand the relationship between the epigenome and antigen-specific antibody responses to pneumococcal vaccination.

**Methods:**

The epigenetic profiles from 24 healthy children were analyzed at 12 months prior to a booster dose of the 13-valent pneumococcal conjugate vaccine (PCV-13), and at 24 months of age, using the Illumina Methylation 450 K assay and assessed for differences over time and between high and low vaccine responders.

**Results:**

Our analysis revealed 721 significantly differentially methylated positions between 12 and 24 months (FDR < 0.01), with significant enrichment in pathways involved in the regulation of cell–cell adhesion and T cell activation. Comparing high and low vaccine responders, we identified differentially methylated CpG sites (*P* value < 0.01) associated with *HLA-DPB1* and *IL6*.

**Conclusion:**

These data imply that epigenetic changes that occur during early childhood may be associated with antigen-specific antibody responses to pneumococcal vaccines.

## Background

Young children are at higher risk of developing life-threatening diseases compared with adults, which is thought to be due to the immaturity of their immune system. They also show reduced immune responses to vaccines [1]. Pneumococcal infections are a major public health problem worldwide causing high morbidity and mortality in young children who suffer pneumonia, meningitis and septicemia, leading up to 1 million deaths in children under the age of five years [2, 3]. Despite many years of research, the specific characteristics of the immune system in early childhood that underpins increased susceptibility to infection has still not been fully elucidated [4]. Furthermore, there is great variability in the immune response to vaccination in children, with some individuals achieving more than 100-fold higher antibody levels post-immunization than others [5, 6]. The reason for this huge variation is not understood, and recent studies have begun to reveal the genetic determinants of immune responses to vaccines [7, 8].

The term epigenetics or ‘the epigenome’ describes the summary of heritable changes in expression (i.e., function) of genes that are not encoded in the DNA sequence and form a mechanism whereby past environmental events may be encoded and modify future cellular responses. Epigenetic mechanisms have repressive and permissive effects on gene expression and thus play a key role in cellular differentiation. DNA methylation forms the archetypal, heritable, epigenetic mark and involves the chemical modification of cytosine and cytosine:guanine dinucleotides, resulting in profound, typically repressive, effects [9]. DNA methylation patterns can be measured across the whole genome covering 99% of currently annotated genes in a high-throughput manner [10]. They differ between individuals, even between identical twins, and their change over time is influenced by intrauterine and postnatal environmental factors such as nutrition, toxins or drugs and illnesses [11]. Recent studies have demonstrated that the levels of DNA methylation of certain CpG sites are also highly correlated with human chronological age [12]. Moreover, these DNA methylation features have been proposed as a surrogate of the “biological age,” which may actually be more relevant than chronological age in predicting age-related health risks [13]. Different conditions have been reported to accelerate age-associated DNA methylation profiles such as HIV infection and Down syndrome [14, 15].

There is increasing evidence demonstrating a key role of epigenetics in regulation of human immunity in health and disease [16, 17]. Consistent with this, DNA methylation changes during early childhood have been observed in genes implicated in inflammatory processes, encoded histone modifiers and chromatin remodeling factors [18]. However, the effects of these changes on the immune system are unknown. Importantly, there is limited information on the relationship between epigenetic changes in infancy and the response to vaccination. Here, we sought to investigate epigenetic changes in early life in a well-studied cohort of healthy children, focusing on the characterization of the relationship between epigenetics and age in paired samples collected at 12 and 24 months of age, and the role of epigenetics on the strength of antigen-specific antibody responses to pneumococcal vaccination.

## Results

**Fig. 1:**
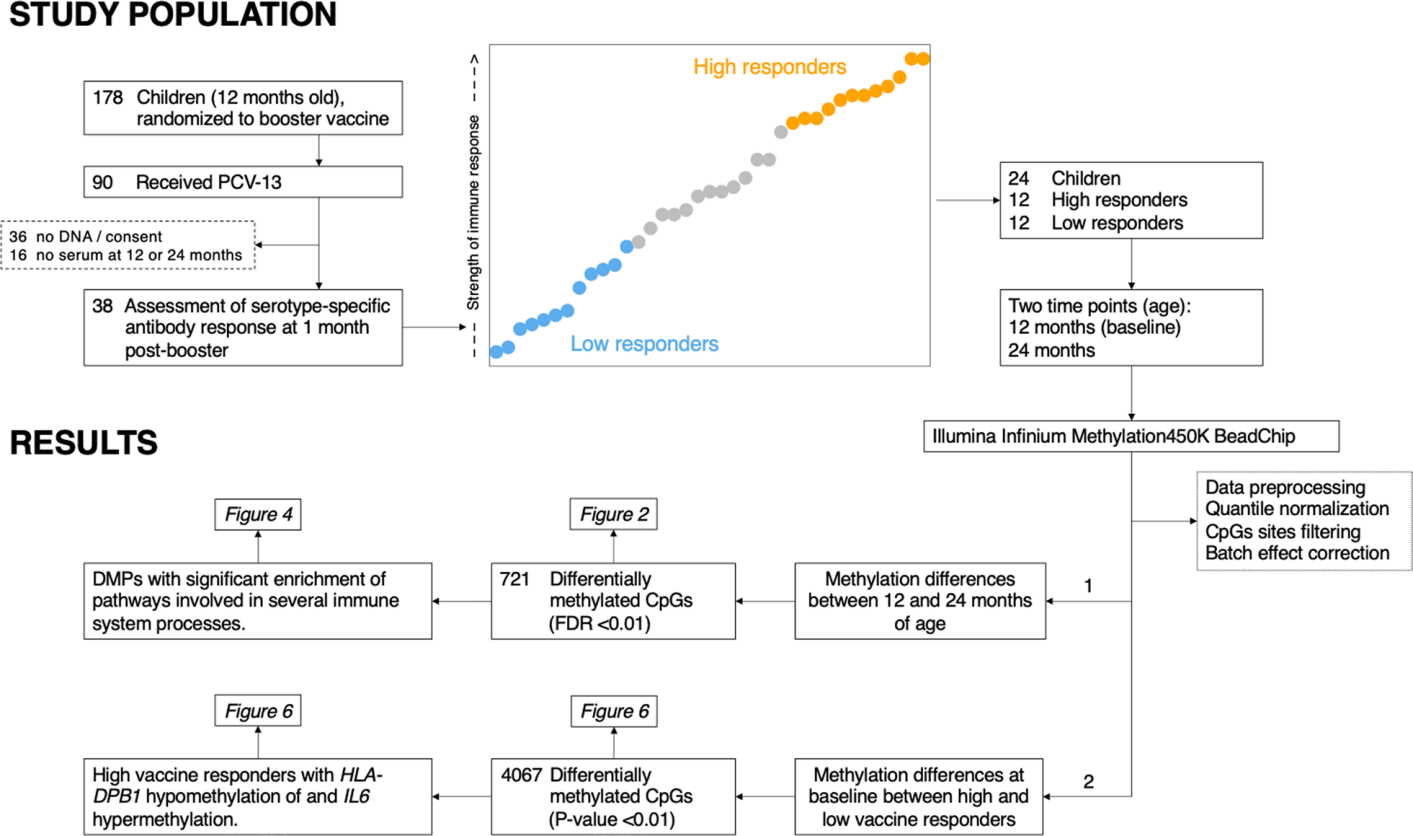
Study design including sample selection for epigenetic studies and overview of main results

Twenty-four children (Table 1) who had received the 13-valent pneumococcal conjugate vaccine (PCV-13) at 12 months of age, as part of a previously published clinical trial [19–21], were selected based on their aggregated IgG antibody response and categorized into high and low responders (Fig. 1 and Additional file 1: Figure 1). Of note, this categorization was also valid for post-booster serotype-specific opsonophagocytic assay (OPA) titers and memory B cells responses (Additional file 1: Figures 2 and 3), therefore truly grouping individuals into high and low vaccine responders. Paired blood samples taken at 12 and 24 months of age were assessed by DNA epigenetic microarray analysis.

**Table 1:** Characteristics of study participants

Pneumococcal vaccine response	Sex	Age at visit 1 (months)	Age at visit 3 (months)	Ethnicity
High	f	12.46	23.82	Indian/British
High	f	12.16	23.46	European/White west African
High	f	12.43	23.72	White Caucasian/European
High	f	12.75	24.05	White Caucasian/European
High	f	12.2	23.98	White Caucasian/European
High	m	12.72	24.57	White Caucasian/European
High	m	11.77	23.52	White Caucasian/European
High	m	12.72	24.25	White Caucasian/European
High	m	13.28	24.61	White Caucasian/European
High	m	12.75	24.28	White Caucasian/European
High	m	12.82	24.61	White Caucasian/European
High	m	12.85	24.08	White Caucasian/European
Low	f	12.56	23.89	White Caucasian/European
Low	f	12.49	23.85	White Caucasian/European
Low	m	12.39	23.95	White Caucasian/European
Low	m	12.16	23.66	Asian South East Asian Heritage
Low	m	12.98	24.74	White Caucasian/European
Low	m	12.39	23.46	White Caucasian/European
Low	m	12.00	23.26	White Caucasian/European
Low	m	13.05	24.38	White South African
Low	m	12.46	23.49	White Caucasian/European
Low	m	12.36	24.21	Canadian/British
Low	m	13.18	24.51	White Caucasian/European
Low	m	12.92	24.9	White Caucasian/European

*f* female, *m* male

### Widespread changes in the epigenome occur between 12 and 24 months of life

**Fig. 2:**
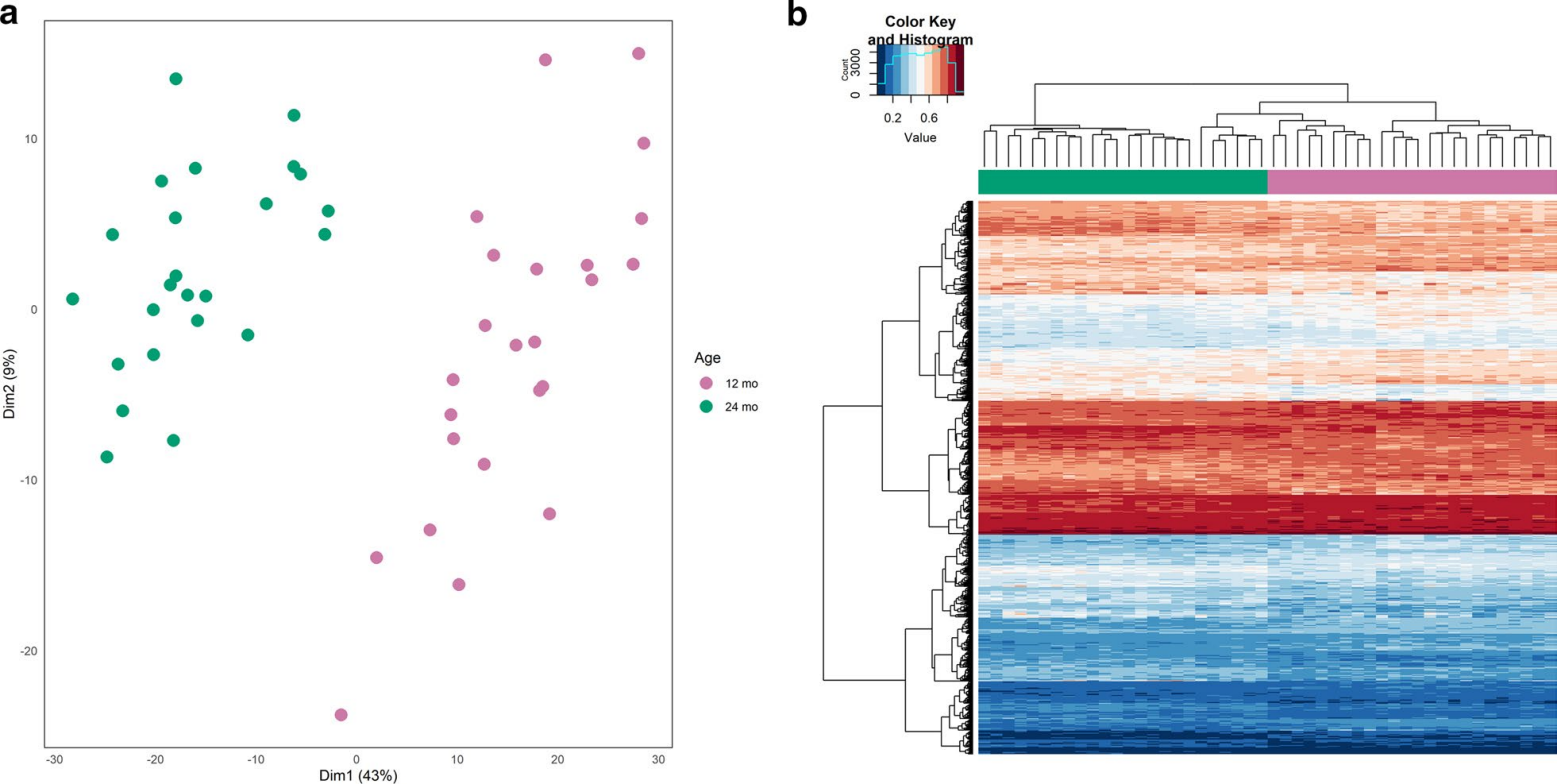
**a** Principal component analysis and **b** heatmap including unsupervised clustering of age (24 vs. 12 months) using the 721 significant differentially methylated positions

The first step of our analysis focused on the methylation changes between the two timepoints (12 vs. 24 months). After correcting our linear model for cell-type composition, we found 721 significant DMPs, associated with 421 unique genes. Considerable inflation could be observed in the QQ plot (Additional file 1: Figure 4), reflecting substantial age-related changes to the blood epigenome early in life. Of the significant DMPs, 314 (43.6%) were hypomethylated with the remaining 407 (56.4%) being hypermethylated, in samples collected at 12 months of age (Additional file 7: Table 1). The results without adjusting for cell compositions are shown in Additional file 8: Table 2. There was a clear separation between samples from 12 and 24 months of age using PCA and hierarchical clustering of the 721 DMPs (Fig. 2).

Further analyzing the 721 DMPs, we observed several DMRs where multiple adjacent CpGs probes were found together showing differential methylation between the two different timepoints. For instance, 10 probes were associated with nuclear factor I X (*NFIX*), and 12 CpGs were observed within the same island region, chr6:32118101–32118544, of the proline-rich transmembrane protein 1 (*PRRT1*). Hypomethylated CpGs were mainly found in CpG islands, whereas open sea, shore and shelf regions were mostly hypermethylated (Additional file 5: Figure 5a). Most DMPs were found in the gene body, TSS1500 and 5′ UTRs and dominated by hypomethylation patterns (Additional file 1: Figure 5b).

We further analyzed DMPs, which showed the highest age-dependent differences in methylation by applying a delta beta (difference in the mean β value of each group) threshold of ≥ 0.10. In this way, we detected 24 DMPs, considered as the DMPs most separating the two age groups (Table 2); of those, 18 CpGs exhibited a higher methylation level at 24 months, while the remaining 6 had a lower methylation pattern at 24 months.

**Table 2:** List of the 24 DMPs with an absolute difference in methylation levels > 0.10 between 12 and 24 months of age

CpG_ID	Chr	Position	Relation to CGI	Gene name	Gene group	FDR *P *value	Mean value (m12–m24)
cg05825244	chr20	2,730,488	Island	*EBF4*	Body	2.96E−04	− 0.2201
cg25556035	chr19	13,127,873	S_Shelf	*NFIX*	Body	4.27E−07	− 0.1930
cg14716990	chr10	129,533,731	N_Shore			9.20E−08	− 0.1523
cg22268510	chr6	32,118,420	Island	*PRRT1*	Body	5.14E−07	− 0.1448
cg10767662	chr19	13,127,729	S_Shelf	*NFIX*	Body	3.43E−07	− 0.1407
cg01323777	chr17	7,832,943	Island	*KCNAB3*	TSS200	4.09E−05	− 0.1366
cg00254681	chr6	32,118,457	Island	*PRRT1*	Body	1.01E−06	− 0.1321
cg13138089	chr2	233,251,770	Island	*ECEL1P2*	TSS200	7.75E−03	− 0.1309
cg13870494	chr9	72,658,358	N_Shore	*MAMDC2*	TSS200	2.36E−05	− 0.1244
cg20471691	chr17	46,681,316	N_Shelf	*LOC404266; LOC404266; HOXB6*	Body; body; 5′UTR	4.05E−05	− 0.1164
cg23365801	chr17	7,832,909	Island	*KCNAB3*	TSS200	8.93E−04	− 0.1163
cg23491743	chr2	241,989,271	Island	*SNED1*	Body	2.15E−06	− 0.1141
cg27162435	chr17	7,833,163	Island	*KCNAB3*	TSS1500	8.86E−05	− 0.1099
cg00589520	chr7	23,513,039	N_Shore			1.15E−09	− 0.1053
cg09490371	chr2	233,253,024	Island	*ECEL1P2*	TSS1500	2.54E−03	− 0.1051
cg11617964	chr6	32,118,399	Island	*PRRT1*	Body	1.82E−08	− 0.1027
cg25460807	chr8	21,908,022	S_Shelf			6.25E−04	− 0.1013
cg11041817	chr17	46,685,327	Island	HOXB7	Body	4.88E−03	− 0.1011
cg16146033	chr11	62,767,323	OpenSea	*SLC22A8*	Body	5.40E−08	0.1063
cg02481642	chr20	43,343,760	OpenSea	*WISP2*	TSS200	5.78E−06	0.1085
cg25135018	chr1	154,435,948	OpenSea	*IL6R; IL6R*	Body; body	1.16E−06	0.1099
cg06688910	chr8	122,466,955	OpenSea			6.72E−08	0.1101
cg17945323	chr11	62,767,406	OpenSea	*SLC22A8*	Body	6.66E−09	0.1103
cg09978533	chr22	46,465,160	N_Shore			9.39E−06	0.1149

The top 18 CpGs are hypermethylated, while the bottom 6 CpGs are hypomethylated in samples from 24 months compared with 12 months of age

*Chr* chromosome, *N_Shore/N_Shel North Shore/Shelf, *S_Shore/S_Shelf* South Shore/Shelf

### Age-associated changes in the epigenome are enriched for pathways linked to T cell regulation and activation

**Fig. 3:**
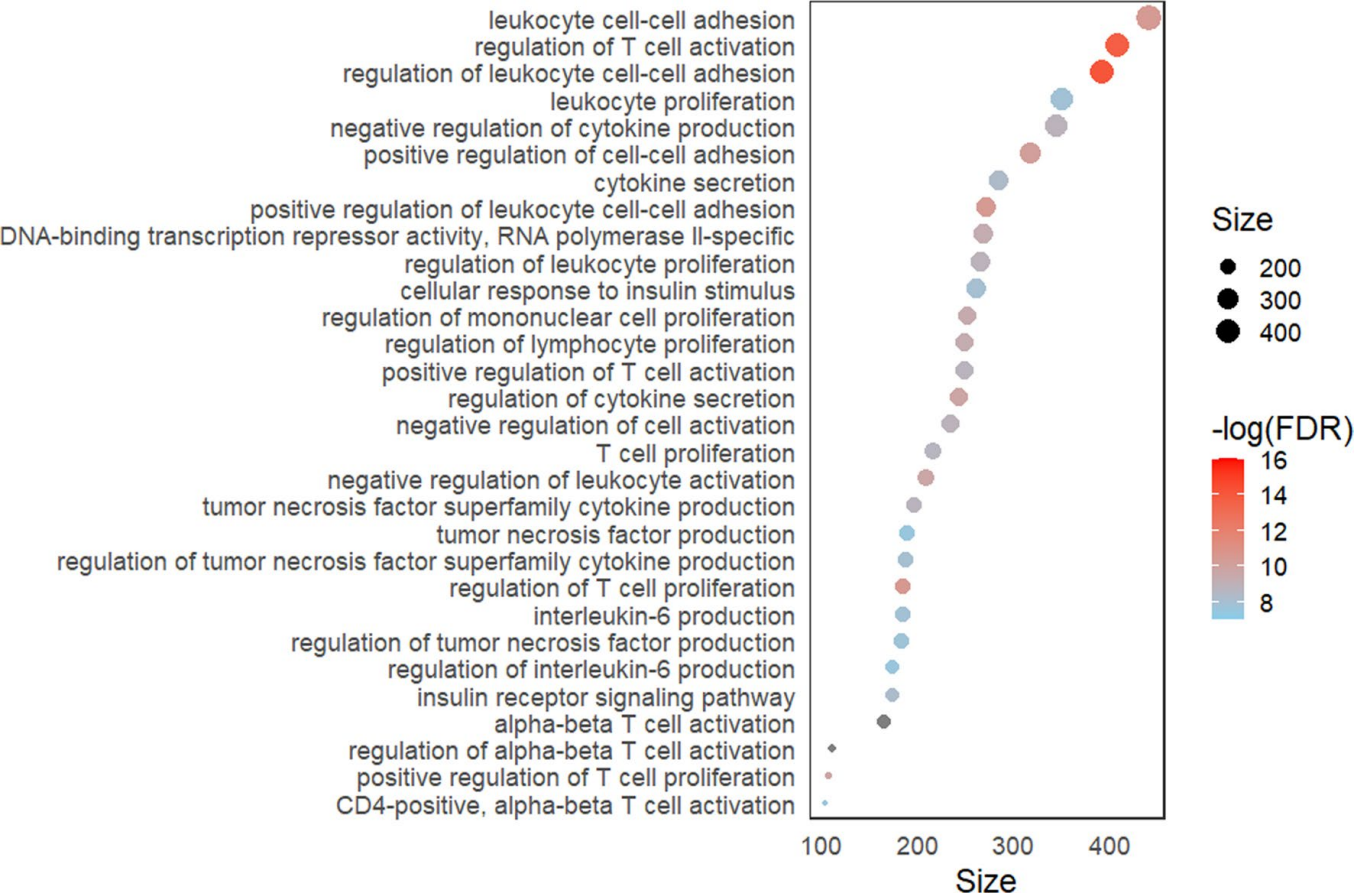
Dot plot of the top 30 GO pathways (FDR < 0.05). Size along the *x*-axis indicates the number of genes involved in each pathway. Dot colors correspond to the different FDR *P* values associated with the pathways

To investigate which molecular functions or biological processes were associated with the DMPs between 12 and 24 months of age, gene set enrichment analysis was performed. We detected significant enrichment (FDR < 0.05) in 151 GO pathways, with the most significant pathways being related to regulation of T cell activation, cell–cell adhesion and regulation of cytokine production (Fig. 3; Additional file 9: Table 3).

**Fig. 4:**
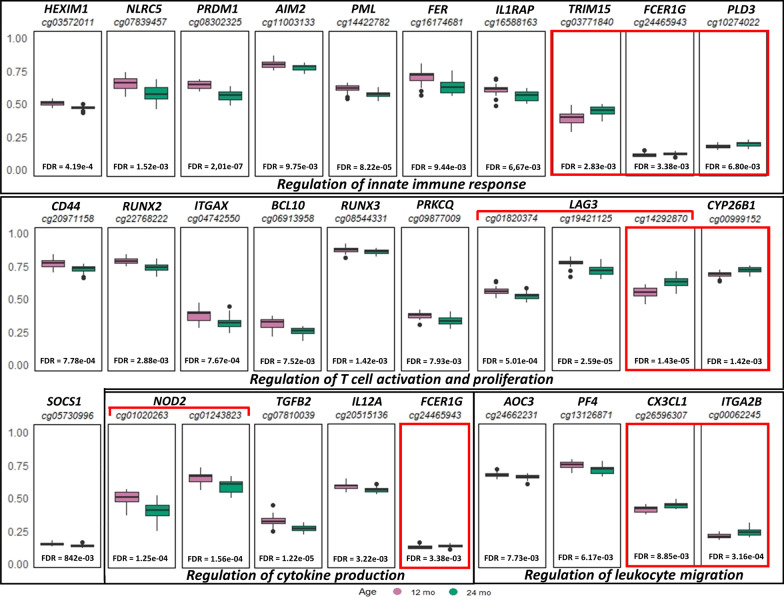
Thirty of the most significant DMPs associated with immune genes grouped according to their GO function. The *y*-axis represents the *β*-value for each position (purple for 12 months, green for 24 months). Red squares indicate CpGs with hypermethylation at 24 compared with 12 months samples, while for the remaining CPGs, hypomethylation was found at 24 compared with 12 months of age

Among DMPs, we observed 86 CpGs associated with 69 unique genes within immune system processes according to GO terms. Of these, 53 CpGs showed a decrease in methylation with age, while the other 32 exhibited an increase in methylation from 12 to 24 months (Fig. 4). Some of the genes were represented by multiple DMPs, such as *HOXB6*, *NOD2* and *LAG3*, in the latter we found both hypo- and hypermethylated CpGs*.*

**Fig. 5:**
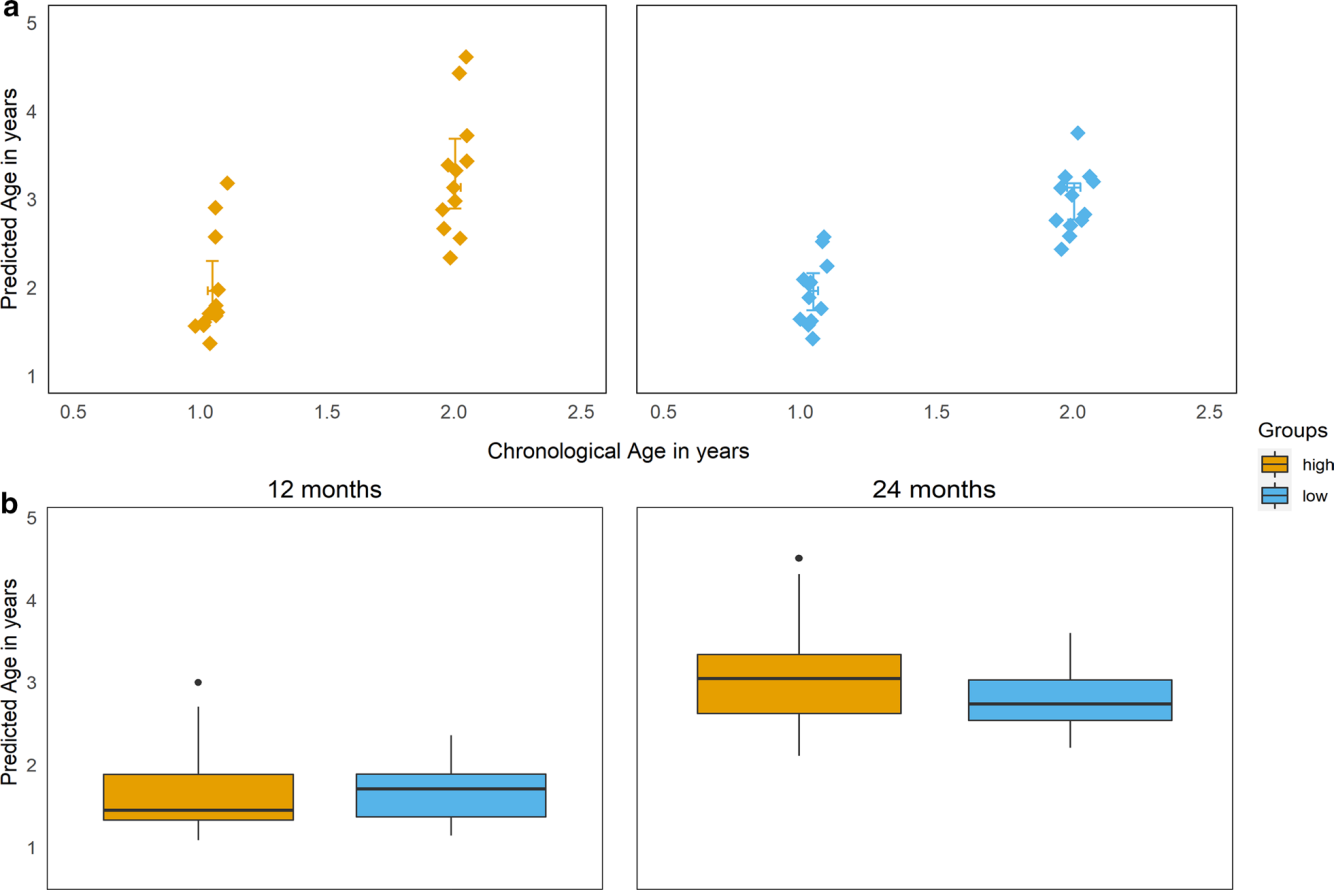
**a** Correlation between chronological (*x* axis) and predicted biological age (y axis) as calculated with the Horvath epigenetic age estimator. **b** Comparison of predicted biological age between high- and low-responders at the two study time points

In order to understand if the different responsiveness to vaccination was associated with the “biological age” of the subjects, we used the epigenetic age estimator developed by Horvath in 2013 [22] to calculate biological age based on methylation patterns. First, we applied age predictors to the results obtained from samples at the two time points and found that chronological and predicted biological ages were correlated (Fig. 5a). We did not observe significant differences in predicted biological age between high and low vaccine responders (Fig. 5b).

### Hypomethylation of HLA-DPB1 and hypermethylation of IL-6 is associated with more robust responses to pneumococcal infant vaccination

**Fig. 6:**
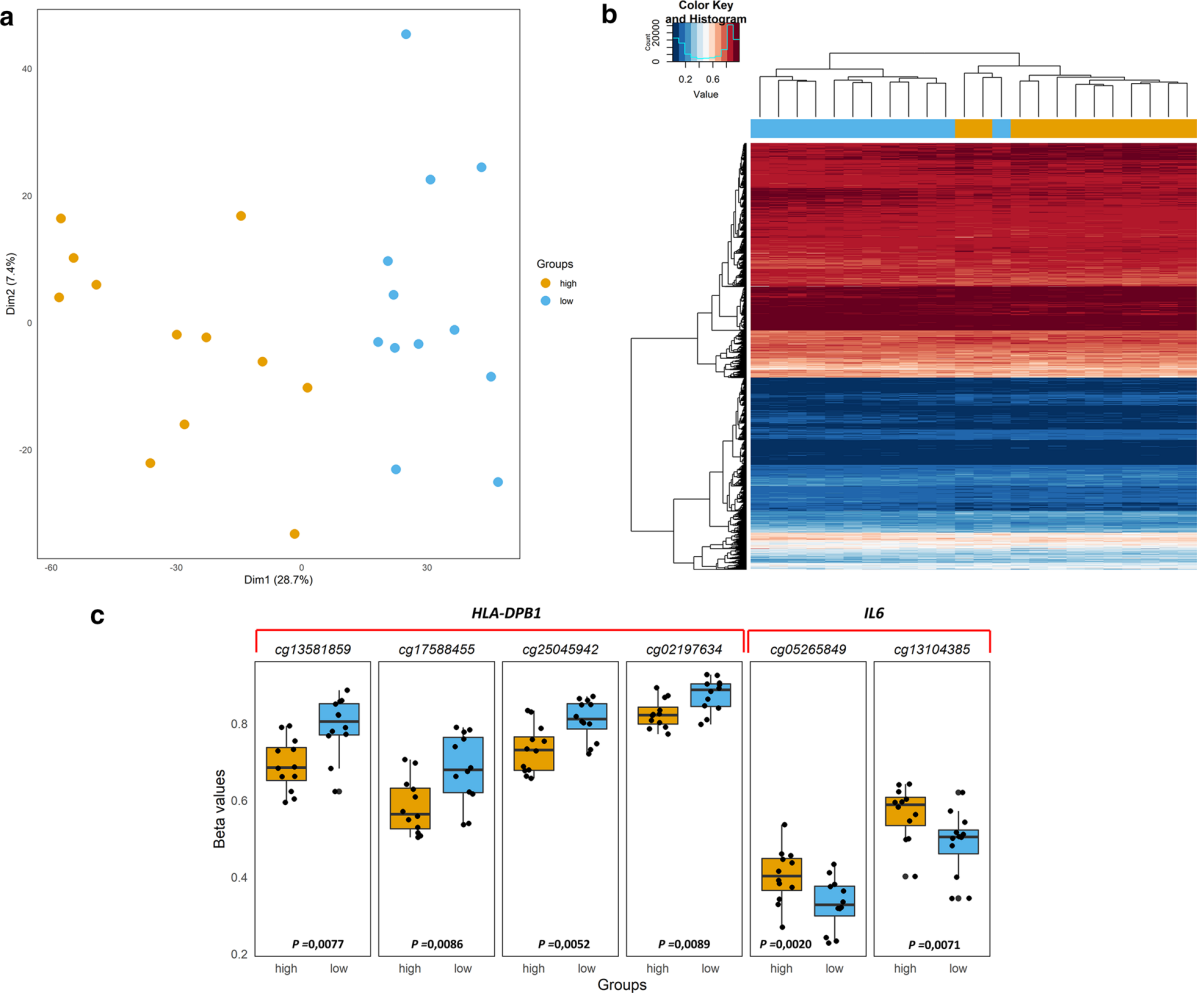
**a** Principal components analysis and **b** unsupervised clustering with heatmap of 4067 differentially methylated CpG sites (uncorrected *P* value < 0.01) at 12 months of age and grouping by high and low vaccine responders. **c** Boxplots showing 4 DMPs associated with hypomethylation of *HLA-DPB1* and 2 DMPs associated with hypermethylation of *IL6* in high vaccine responders. The *y*-axis represents the *β* value for each position in both groups (yellow for high responders, light blue for low responders)

We next assessed whether differences in antibody responses against vaccine antigens were associated with methylation patterns by comparing pre-vaccination blood epigenome profiles of high responders to those of low responders. After applying a FDR < 0.01 correction, no statistically significant DMPs were detected. However, using a less stringent statistical threshold (uncorrected *P* value < 0.01), we found 4067 differentially methylated CpG sites (Additional file 10: Table 4) associated with 2797 unique genes that distinguished high from low responders in a PCA (Fig. 6a) and an unsupervised clustering method (Fig. 6b). Similar results were obtained for a group comparison at 24 months (Additional file 6: Figure 6).

We further investigated among the 4067 DMPs those which showed the highest difference in methylation between high and low vaccine responders (mean β value) and assessed regions containing multiple DMPs in genes involved in immune system processes. Among the top DMPs identified, there were 4 hypomethylated positions in high vaccine responders within the chr6:33048416–33048814 island region of the HLA class II histocompatibility antigen DP(W2) beta chain (*HLA-DPB1*). There were also two CpGs associated with interleukin 6 (*IL-6*) that showed hypermethylation in high compared with low vaccine responders (Fig. 6c).

## Discussion

In the present study, we demonstrated age-associated DNA methylation changes between the first and second year of life that were largely related to T cell regulation and activation. In addition, using detailed antibody measurements following infant pneumococcal conjugate booster vaccination in a clinical vaccine trial, we found hypomethylation of *HLA-DPB1* and hypermethylation of *IL6* to be correlated with a stronger response to pneumococcal vaccination.

A total of 721 CpGs were found to be significantly differentially methylated between 12 and 24 months of age. Multiple DMPs within several DMRs showed similar methylation patterns suggesting a general epigenetic remodeling of these genetic loci. For example, within the chr6:32118101–32118544 locus, there were twelve DMPs that exhibited a significantly higher methylation pattern at 24 compared with 12 months of age. These DMPs are localized within the body of *PRRT1* and could potentially alter the expression of *PRRT1*, as previously demonstrated for other hypermethylated positions in genes outside of the promoter area [23]. Little is known about the function of *PRRT1* although aberrant methylation of this gene has been related to neurodevelopmental disorders [24] and to hepatic tumorigenesis [25]. Furthermore, we observed a total of 118 CpGs that were previously reported to be altered between 3 and 60 months in blood leukocytes [18]. In agreement with this previous work, we observed 57 CpGs exhibiting an increase in methylation between 12 and 24 months of age, and 60 CpGs showing a decreased methylation with age [18].

Through an enrichment pathway analysis of the age dependent DMPs, we observed 86 CpGs that were associated with 69 genes involved in several immune system processes. Genes that were hypomethylated at 24 compared with 12 months included *IL12A*, previously shown to be repressed at chromatin level in neonatal mononuclear compared with adult cells [26–28], as well as *BCL10* and *NOD2*, also previously demonstrated to decrease methylation with age [18]. In contrast, CpGs with an increase in methylation between 12 and 24 months included genes involved in the regulation of innate immune responses such as tripartite motif containing 15 (*TRIM15*) [30], and the PML Nuclear Body Scaffold (*PML*) [31]. A particular pattern of methylation was observed for lymphocyte activating 3 (*LAG3),* also required for T cell regulation [32]. *LAG3* was hypomethylated at two positions within the chr12:6882855–6883184 region and hypermethylated in an open sea CpG at 24 months, suggesting that diverging methylation patterns may regulate expression of this gene.

There was a clear separation of predicted biological age between samples taken at the two study time points. Predicted biological age was moderately higher than chronological age, most likely due to the small range of errors for whole blood samples that the age predictor exhibits [22]. However, predicted biological age was not significantly different between high and low vaccine responders at any time point suggesting no differences attributable to “epigenetic age” between the groups.

The second part of our research study focused on the relationship between the epigenome at 12 months of age before immunization and the strength of the antigen-specific IgG response to vaccination. We identified a high number of CpGs that separated high from low vaccine responders using a less stringent statistical threshold (uncorrected *P* value < 0.01). These CpGs included multiple probes mapping to genes involved in immune system processes and were associated with large differences in methylation between the two groups. In this way, we observed two particularly interesting immune genes (*HLA-DPB1* and *IL6*) that contained multiple DMPs differing between high and low vaccine responders. Hypomethylation of *HLA-DPB1* as found in high compared with low vaccine responders may indeed strengthen the overall immune response as previously demonstrated by the contribution of HLA to cellular host immunity in response to anthrax vaccine adsorbed [33]; rubella vaccine [34]; hepatitis B vaccine; and the inactivated Japanese encephalitis vaccine [35]. Additional evidence connecting HLA genotype and expression to vaccine immune responses was found in measles seropositive and seronegative individuals [36]. Two CpGs in the body of *IL6* showed higher methylation in high compared with low vaccine responders. These CpGs in open sea positions (i.e., isolated in the genome [37]) are considered to be “predicted enhancer elements” [38], therefore possibly impacting *IL6* expression. Il-6 is a proinflammatory cytokine involved in a variety of immune processes and also plays an important role in mediating innate and adaptive immune responses [39]; therefore, differential expression of *IL6* may impact vaccine immune responses. However, in contrast to our finding of hypermethylated (and possibly reduced expression of) *IL6* in high vaccine responders, previous work suggested that IL-6 promotes stimulation of humoral and cellular immune responses [40–43].

There are several limitations to our study. First, several samples had to be excluded due to unavailability of genetic material, consent for genetic analysis or missing serum, which may have affected the results. In addition, high and low responders were categorized using the aggregated serotype-specific IgG response 1-month post-booster vaccination, which may not accurately reflect the strength of the overall immune response to immunization. Reassuringly, however, high vaccine responders showed similar patterns in OPA and memory B-cell responses, indicating that this categorization also seems to hold up in different measurements of the immune response.

## Conclusion

In the present study, we have studied changes in methylation patterns of healthy children between 12 and 24 months of age and found that these involved regions dominated by immune pathways such as T cell regulation and activation. In addition, we found a number of CpGs associated with the strength of the antigen-specific IgG antibody response to a pneumococcal conjugate booster vaccine including differences in methylation patterns in *HLA-DPB1* and *IL6*. These latter findings suggest that epigenetic patterns may influence antibody responses in young children and may potentially add to their susceptibility to infection.

### Methods

#### Study subjects and sample selection

In a previous clinical trial, 178 children were randomized to receive a booster dose of either the 10- or the 13-valent pneumococcal conjugate vaccine (PCV-13) at 12 months of age following receipt of PCV-13 at 2 and 4 months of age [19–21]. Ethical approval for this clinical vaccine trial was obtained from the Oxfordshire Research Ethics Committee (Reference number 11/SC/0473). Ethical permission to store and study genetic material was obtained prior to enrolment into the study, in accordance with the Oxford Vaccine Centre Biobank (10/H0504/25). Detailed immune responses were measured before and at 1 and 12 months after the booster. At the three study time points, anti-polysaccharide serum Immunoglobulin G (IgG) and opsonophagocytic assay (OPA) titers to all PCV-13 serotypes were determined, and antigen-specific memory B cell responses were measured for selected pneumococcal polysaccharides. For the purpose of this study, 24 individuals who had received a PCV-13 booster dose at 12 months of age (*n* = 90) (baseline group) were selected based on their aggregated IgG antibody response and categorized into high and low responders. For this categorization, the strength of the polysaccharide-specific IgG response was ranked for each PCV-13 recipient and vaccine serotype at 1 month following the booster. The overall IgG antibody response was then calculated by determining the median rank for all serotypes. After excluding participants with missing serum or DNA samples, 38 individuals remained, of whom the 12 high and 12 low responders were selected (Fig. 1). This categorization was also valid for post-booster serotype-specific OPA and memory B cells responses. We used Infinium Methylation 450 K to profile (1) the epigenetic differences between two timepoints: 12 months (baseline group) and 24 months and (2) to elucidate the methylation differences between high and low PCV-13 booster vaccine responders, in order to identify pathways that show significant changes over time and those that are associated with a more robust immune response.

### Laboratory procedures

Polysaccharide-specific antibody measurements were performed as previously described [19, 44]. DNA was extracted from blood clots collected from the same participants at 12 and 24 months of age. Whole blood DNA was bisulfite converted using the EZ-96 DNA Bisulfite Zymo Research conversion protocol according to the manufacturer’s instructions. The treated DNA was then hybridized to the Illumina Infinium Methylation450K BeadChip, and the array was imaged using the Illumina iScan system in which the percent methylation state of each CpG site was quantified for the entire study group.

### Data analysis

The epigenetic profile from study participants was measured at both time points using the Illumina Methylation 450 K assay covering > 485,000 methylation sites per sample at single-nucleotide resolution. DNA methylation analyses were conducted using R software (v.3.6.1) and several Bioconductor packages. Raw intensity files were preprocessed and transformed into β and M values using the R package minfi. The intensities of β values were estimated from the intensity ratio of the methylated signals over the total (methylated and unmethylated) signals for each site that represents the percentage of methylation at a given cytosine for an individual across the blood cells. The β value ranges between 0 (position not methylated in any of the cells) and 1 (position methylated in all cells in the sample). The M value instead is defined as the log2 ratio of the intensities of methylated vs. unmethylated probes and indicates hypermethylation (if positive) and hypomethylation (if negative). M values close to 0 represent a similar intensity between methylated and unmethylated probes.

All raw data passed quality control and normalized using the function preprocessQuantile of the minfi package. Subsequently, to avoid artifactual data, probes with a detection *P* value > 0.01, probes located in sex chromosomes, probes in sites containing SNPs or with a minor allele frequency < 5% and probes known to have cross-reaction were removed. This filtering removed 28,999 leaving 456,513 probes for subsequent analysis. As the DNA samples were obtained from peripheral blood and because of the extreme cell-type specificity of DNA methylation [45], cell composition heterogeneity was evaluated [46] using the package FlowSorted.Blood.450 K and the estimateCellCounts function. Batch effect was removed using Combat from SVA package.

Identification of differentially methylated probes/positions (DMPs) between the study timepoint and high- and low-responders to the vaccine was performed using limma package [47] fitting a linear regression model to each CpGs site. M-values are more homoscedastic than β-values and were thus used for all statistical analyses because of their better detection sensitivity [48]. However, we report β-values in text and figures to facilitate interpretation. Through limma package, it was possible to include covariates in the specification of the model (M values ~ Group + Individuals + Responder + Sex + Cell Composition). Output of the linear model was further analyzed using an empirical Bayes method to moderate standard errors. *P* values were corrected for multiple testing using the Benjamini–Hochberg false discovery rate (FDR) method [49], and DMPs were defined as all positions with an FDR *P* value < 0.01. CpGs were annotated using Bioconductor package lluminaHumanMethylation450kanno.ilmn12.hg19 (v1.2) to genomic regions classified in regions 200 bp upstream of the transcription start site (TSS; TSS200), 1500 bp upstream of the TSS (TSS1500), the 5′ and 3′ untranslated regions (UTRs), 1^st^ exon and gene body. Regions are further subdivided according to their relation to CpG-rich DNA sequences, the so-called CpG islands (CGI), in CpG N or S shelves (within ~ 4 kb north or south from CGI), CpG N or S shores (within ~ 2 kb north or south from CGI) and open sea (region > 4 kb from CGI). Differentially methylated regions (DMRs) refer to genomic regions with multiple differentially methylated positions close together and with different methylation patterns across samples.

Principal component analysis (PCA) was performed to visualize global epigenome patterns comparing 12- and 24-month samples as well as samples from high and low responders. Pathway analysis was performed employing the methylglm function of the methylGSA package [50]. This function adjusts for the number of CpGs by implementing a logistic regression model using a functional class scoring method; all CpGs ranked by differential methylation test *P* values were considered in gene set testing since each CpGs could be of interest. Pathways enrichment was considered significant if false discovery rate (FDR) < 0.05.

## Supplementary Information

**Additional file 1: Figure 1**. Boxplots of IgG antibody concentrations against PCV-13 serotypes (serotype indicated above each box) in low and high responders (n = 12 each; based on aggregated IgG antibody concentration 1 month post-vaccination) as well as middle responders (n = 14) and children without available DNA (n = 36).

**Additional file 2: Figure 2**. Boxplots of OPA antibody titers against PCV-13 serotypes (serotype indicated above each box) in low and high responders (n = 12 each; based on aggregated IgG antibody concentration 1 month post-vaccination) as well as middle responders (n = 14) and children without available DNA (n = 36).

**Additional file 3: Figure 3**. Boxplots of IgG antibody-secreting cell frequencies against selected PCV-13 serotypes (serotype indicated above each box) in low and high responders (n = 12 each; based on aggregated IgG antibody concentration 1 month post-vaccination) as well as middle responders (n = 14) and children without available DNA (n = 36).

**Additional file 4**. QQ plot of the observed* p*-values against the expected* p*-values.

**Additional file 5**. Distributions of the 721 age-dependent DMPs (a) in the island context (island, shore, shelf, open sea) and (b) in the genomic regions (1st exon, 3’ UTR, 5’ UTR, body, TSS1500, TSS200).

**Additional file 6**. (a) Principal component analysis and (b) unsupervised clustering and heatmap of 5233 differentially methylated CpG sites detected using a threshold of an uncorrected* p*-value < 0.01 at 24 months of age and grouping by high and low vaccine responders.

**Additional file 7: Table 1**. Significantly differentially methylated positions (FDR adjusted p-value <0.01) between 12 and 24 months of age, adjusted for individual, responder, gender and cell composition.

**Additional file 8: Table 2**. Differentially methylated positions found to be significant (FDR adjusted* p*-value < 0.01) between 12- and 24-months groups, without adjusting the model for cell composition.

**Additional file 9: Table 3**. List of 152 significant enriched pathways in GO (FDR < 0.05).

**Additional file 10: Table 4**. Differentially methylated positions (*p*-value < 0.01) found to be differentially methylated between high and low responders at 12 months of age, adjusted for gender and cell composition.

## Data Availability

Methylation data have been deposited on the Gene Expression Omnibus (GSE165170; https://www.ncbi.nlm.nih.gov/geo/).
