# Supplementary material for: Changes in epigenetic profiles throughout early childhood and their relationship to the response to pneumococcal vaccination
Source: Clin Epigenetics. 2021 Feb 4;13:29. doi: 10.1186/s13148-021-01012-w (PMC7860179; doi:10.1186/s13148-021-01012-w)
Supplement: Supplementary file 2 — Additional file 2: Figure 2. Boxplots of OPA antibody titers against PCV-13 serotypes (serotype indicated above each box) in low and high responders (n = 12 each; based on aggregated IgG antibody concentration 1 month post-vaccination) as well as middle responders (n = 14) and children without available DNA (n = 36). [file 13148_2021_1012_MOESM2_ESM.pdf]

Figure 2

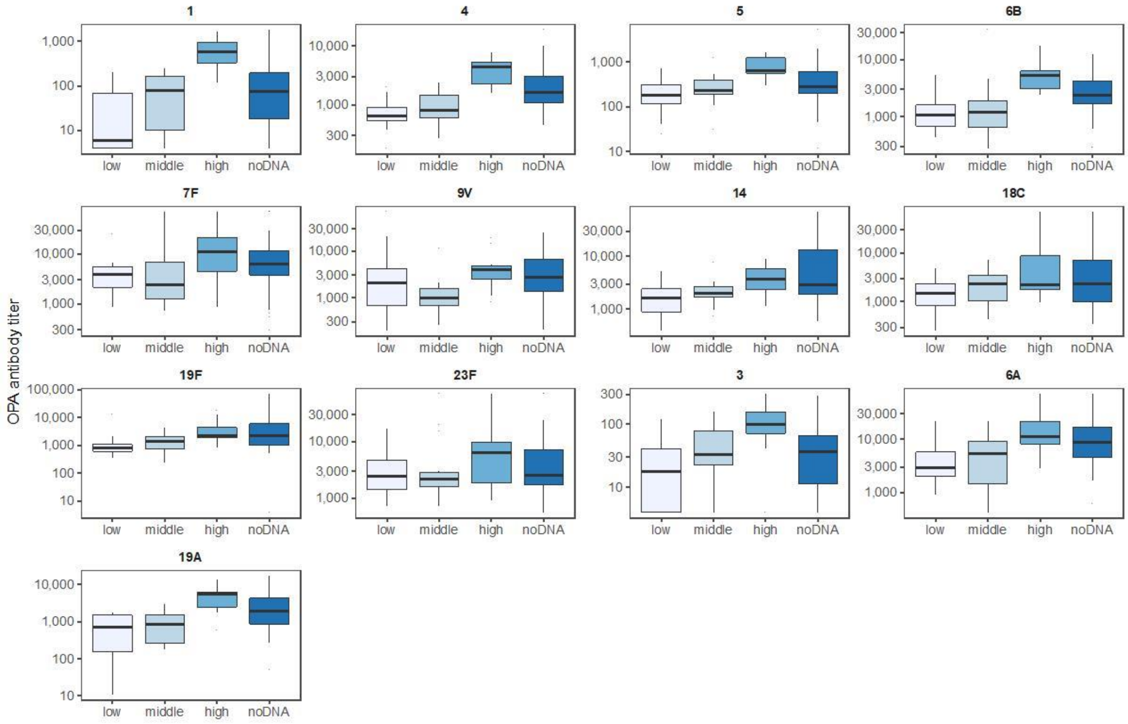

Boxplots of OPA antibody titers against PCV-13 serotypes (serotype indicated above each box) in low and high responders (n = 12 each; based on aggregated IgG antibody concentration 1 month post-vaccination) as well as middle responders (n = 14) and children without available DNA (n = 36).
