# Supplementary figures and images for: Changes in epigenetic profiles throughout early childhood and their relationship to the response to pneumococcal vaccination
Source: Clin Epigenetics. 2021 Feb 4;13:29. doi: 10.1186/s13148-021-01012-w (PMC7860179; doi:10.1186/s13148-021-01012-w)

Figure 4

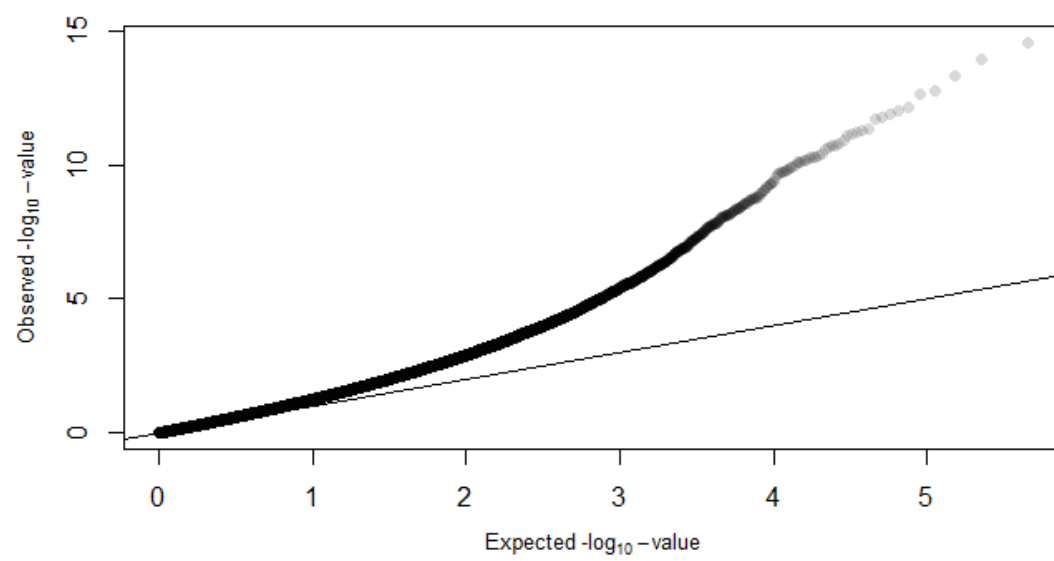

QQ plot of the observed  $p$ -values against the expected  $p$ -values.

Supplement: Supplementary file 4 — Additional file 4. QQ plot of the observed p-values against the expected p-values. [file 13148_2021_1012_MOESM4_ESM.pdf]
