# Supplementary material for: Changes in epigenetic profiles throughout early childhood and their relationship to the response to pneumococcal vaccination
Source: Clin Epigenetics. 2021 Feb 4;13:29. doi: 10.1186/s13148-021-01012-w (PMC7860179; doi:10.1186/s13148-021-01012-w)
Supplement: Supplementary file 5 — Additional file 5. Distributions of the 721 age-dependent DMPs (a) in the island context (island, shore, shelf, open sea) and (b) in the genomic regions (1st exon, 3’ UTR, 5’ UTR, body, TSS1500, TSS200). [file 13148_2021_1012_MOESM5_ESM.pdf]

Figure 5

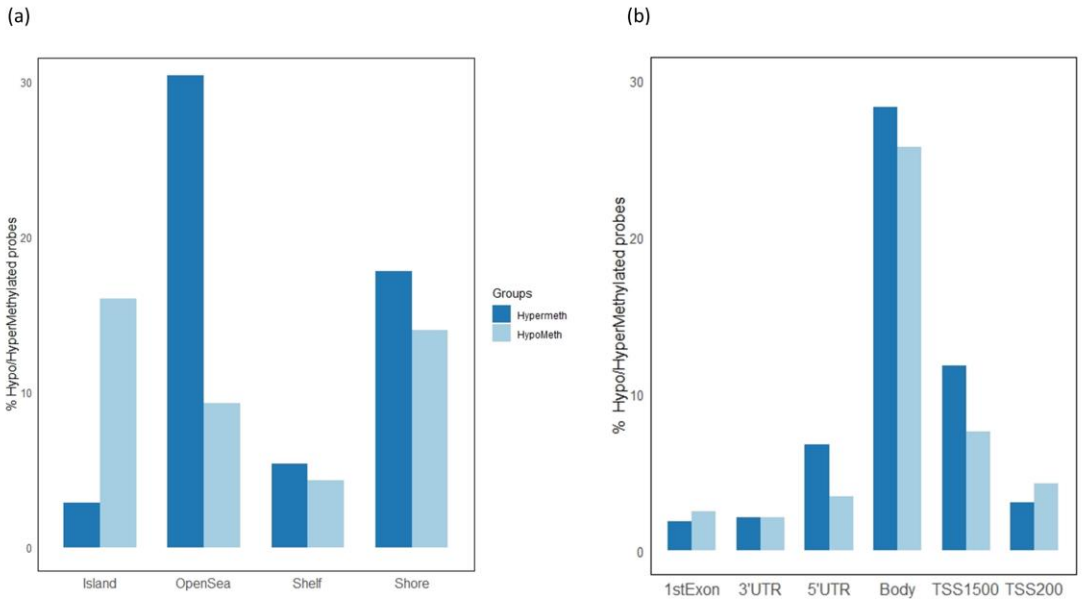

Distributions of the 721 age-dependent DMPs (a) in the island context (island, shore, shelf, open sea) and (b) in the genomic regions (1st exon, 3’ UTR, 5’ UTR, body, TSS1500, TSS200)
