# Supplementary material for: Changes in epigenetic profiles throughout early childhood and their relationship to the response to pneumococcal vaccination
Source: Clin Epigenetics. 2021 Feb 4;13:29. doi: 10.1186/s13148-021-01012-w (PMC7860179; doi:10.1186/s13148-021-01012-w)
Supplement: Supplementary file 6 — Additional file 6. (a) Principal component analysis and (b) unsupervised clustering and heatmap of 5233 differentially methylated CpG sites detected using a threshold of an uncorrected p-value < 0.01 at 24 months of age and grouping by high and low vaccine responders. [file 13148_2021_1012_MOESM6_ESM.pdf]

Figure 6

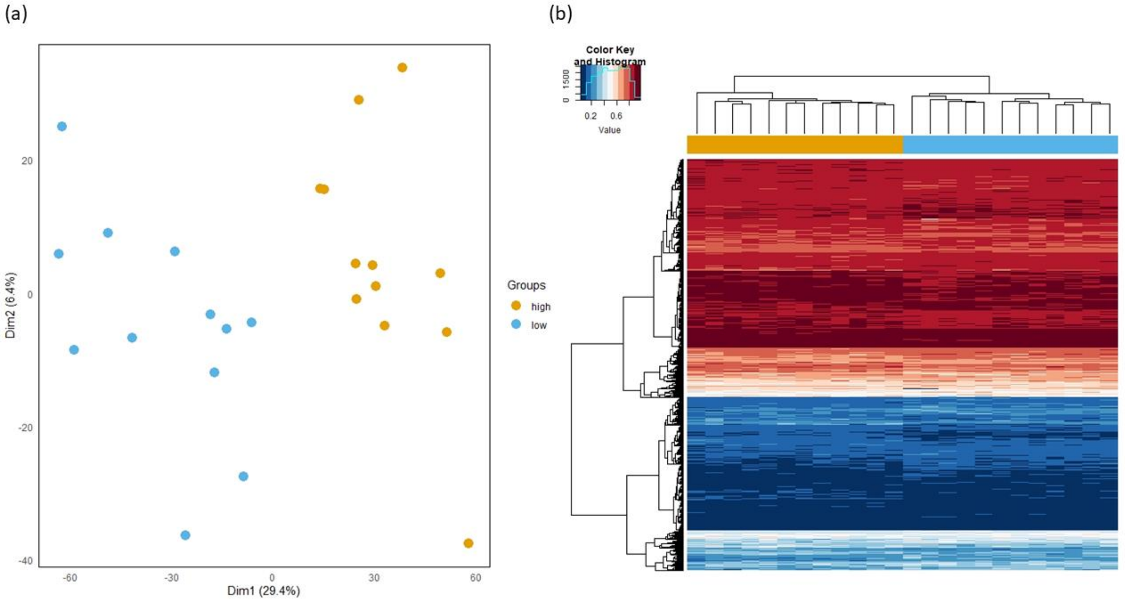

(a) Principal component analysis and (b) unsupervised clustering and heatmap of 5233 differentially methylated CpG sites detected using a threshold of an uncorrected *p*-value <0.01 at 24 months of age and grouping by high and low vaccine responders.
